# Indoor household residual spraying program performance in Matabeleland South province, Zimbabwe: 2011 to 2012; a descriptive cross-sectional study

**DOI:** 10.11604/pamj.2015.20.27.4721

**Published:** 2015-01-12

**Authors:** Pugie Tawanda Chimberengwa, Nyasha Masuka, Notion Tafara Gombe, Mufuta Tshimanga, Lucia Takundwa, Donewell Bangure

**Affiliations:** 1 Department of Community Medicine, University of Zimbabwe, Harare, Zimbabwe; 2 Provincial Medical Director, Ministry of Health and Child Welfare, Matabeleland North Province, Mhlahlandlela Building, PO Box 441, Bulawayo, Zimbabwe

**Keywords:** Matebeleland South, indoor household residual spraying, malaria, pre-elimination

## Abstract

**Introduction:**

Matabeleland South launched the malaria pre-elimination campaign in 2012 but provincial spraying coverage has failed to attain 95% target, with some districts still encountering malaria outbreaks. A study was conducted to evaluate program performance against achieving malaria pre-elimination.

**Methods:**

A descriptive cross sectional study was done in 5 districts carrying out IRS using the logical framework involving inputs, process, outputs and outcome evaluation. Health workers recruited into the study included direct program implementers, district and provincial program managers. An interviewer administered questionnaire, checklists, key informant interviewer guide and desk review of records were used to collect data.

**Results:**

We enrolled 37 primary respondents and 5 key informants. Pre-elimination, Epidemic Preparedness and Response plans were absent in all districts. Shortages of inputs were reported by 97% of respondents, with districts receiving 80% of requested budget. Insecticides were procured centrally at national level. Spraying started late and districts failed to spray all targeted households by end of December. The province is using makeshift camps with inappropriate evaporation ponds where liquid DDT waste is not safely accounted for. The provincial IHRS coverage for 2011 was 84%. Challenges cited included; food shortages for spraymen, late delivery of inputs and poor state of IHRS equipment.

**Conclusion:**

The province has failed to achieve Malaria pre-elimination IRS coverage targets for 2011/12 season. Financial and logistical challenges led to delays in supply of program inputs, recruitment and training of sprayers. The Province should establish camping infrastructure with standard evaporation ponds to minimise contamination of the environment.

## Introduction

Malaria is a disease caused by infection of the red blood cells by a protozoan parasite plasmodium and the vector for inoculation is the female anopheles mosquito [1]. Globally, annual malaria cases range between 300-500 million episodes, leading to more than one million deaths mainly among children under the age of 5 years [2]. The greatest burden of malaria lies within the poor and vulnerable societies [2]. In 2010, World Health Organisation (WHO) estimated 216 million cases of malaria, of which 81% of cases were in Africa [3]. In Zimbabwe, over 50% of the country's population lives in malaria prone areas [4]. Five of the seven districts in Matabeleland South Province are affected by malaria with Beitbridge district having short seasonal malaria while the other four districts have sporadic cases of malaria [4].

The Zimbabwe National Malaria Control Program (NMCP) in 2006 developed a malaria pre-elimination plan for Matabeleland South province as the malaria burden was the lowest nationally [5]. In 2006, the national malaria incidence was 109 per 1000 population while for Matabeleland South Province it was 43 per 1000 population [5]. The malaria incidence for Matabeleland South has been the lowest nationally reaching 17 per 1000 population in 2009 and in 2010 the slide positivity rate for the province was at 20.2% [5].

Vector control is one of the key strategies in malaria control [6]. Recommended vector control strategies are Indoor Household Residual Spraying (IRS), use of Insecticide Treated Nets and Larviciding [6]. IRS is part of Integrated Vector Management strategy which encompasses environmental management, biological control, housing improvement and the use of insecticide treated nets [6]. In Matabeleland South Province, IRS should start from the month of August each year and is supposed to end in December. Pre-elimination activities have been scheduled by the NMCP to start in the year 2012.

Spraying is done in teams comprising 15 spraymen and 8 team leaders. Spraymen are casual semi-skilled workers who are recruited on contract basis from the community and are trained to carry out IRS. Teams are supervised by an IRS coordinator, while a data manager is responsible for accountability of inputs and records mantainence at the IRS camp site.

Worldwide, Dichloro-Diphenyl-Trichoroethane (DDT) has been used for successful eradication of malaria [6]. Previously, DDT was banned as it tends to persist in the ecosystem leading to possible adverse effects on human health [7]. The province has been using pyretroids but with commencement of pre-elimination of malaria phase, DDT has been reintroduced [6]. The use of DDT in malaria elimination is key as long as it is used according to the WHO prescribed statutes [7].

The major objective of IRS is to kill the adult mosquito thereby reducing the mosquito density [6]. In the pre-elimination of malaria, the ideal target is to have 100% coverage of house-holds sprayed [8]. The National Malaria Control Program (NMCP) has set for the nation a target of 95% for the total household sprayed while the percentage population protected by the IRS program is pegged at 85% [4]. The NMCP targets are to reduce the incidence of malaria from 95 per 1000 in 2007 to 45 per 1000 in 2013 while Case fatality rate has to be reduced from 4.5% to 2.5% in 2007 and 2013 respectively [4].

The Africa Malaria Elimination Campaign was launched in 2007 to focus on countries to reduce malaria burden through universal free access to prevention and treatment. In the Africa elimination campaign, Southern Africa Development Community (SADC) identified four countries (Botswana, Namibia, South Africa and Swaziland) with the greatest potential to eliminate malaria by 2015 [9]. Angola, Mozambique, Zambia and Zimbabwe have a relatively high transmission rates, therefore they constitute the second-line countries of the Malaria Elimination [9]. They form a block of 8 countries under the concept of “elimination 8” [9].

IRS has been known to be effective in different epidemiological settings by preventing seasonal increase in transmission, prevention and control of epidemics and it can be used as a tool to eliminate local transmission of malaria [6]. Ultimately, it will reduce malaria prevalence, incidence, morbidity and mortality when applied more regularly and frequently [10]. Monitoring and evaluation is an important aspect where the issues of resistance monitoring and malaria disease surveillance and ability to detect vector resistance need to be planned. Safety of use among spray operators, transportation, storage use and disposal of DDT has to be put in place by a country. Monitoring and evaluation is necessary for evaluation the overall success of the IRS program using DDT [10].

Matabeleland South Province was the first in Zimbabwe to established plans for the pre-elimination of malaria in the year 2006 [8]. The core strategy that is employed is Indoor Residual Spraying where the target for households sprayed is 100 percent. In the 2006-7 spraying season when the pre-elimination plan was conceived, IRS failed to take off in the province, since then to date the percentage spray coverage has failed to attain at least 95 percent target set by the NMCP and a target of 100 percent set by the province [8]. During the period 2006 to 2011, the province still encountered outbreaks of malaria in Beitbridge and Mangwe districts.

We thus posed a question how the Province was performing towards achieving the malaria pre-elimination targets? We therefore conducted this study to evaluate the IRS program to determine its performance for the period 2011 to 2012, in view of achieving targets for malaria pre-elimination that had been commenced in the province. The study recommendations will be used to improve program performance and to assist the province to successfully engage into an effective malaria pre-elimination phase.

## Methods

**Figure 1:**
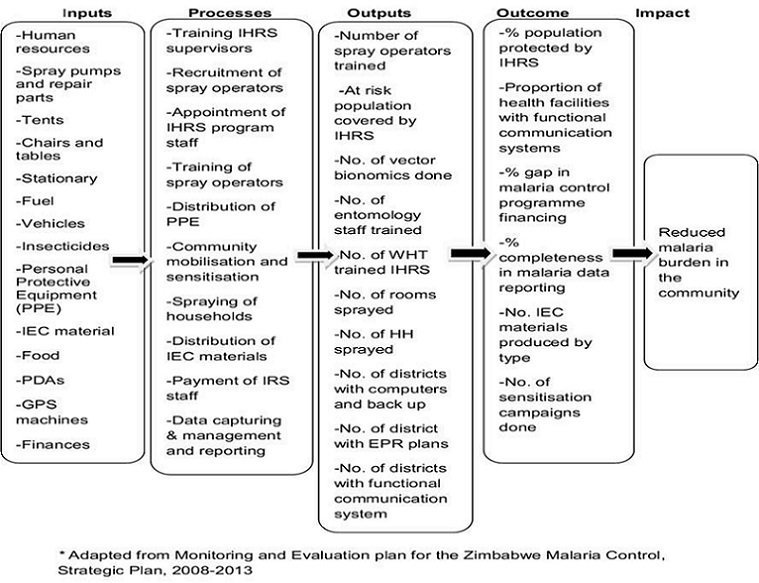
Logic frame work below depicts the inputs processes, outputs and outcome of the Indoor Household Residual Spraying program

**Sampling strategy:** a descriptive cross sectional study was done, using the logical framework (Figure 1), involving inputs, process, outputs and outcome evaluation. The study was carried out in Matabeleland South Province's all 5 districts conducting IRS namely Beitbridge, Bulilima, Gwanda, Mangwe and Matobo. Health workers in the five districts conducting IRS in the province were recruited into the study.

For each district, the direct program implementers (Environmental Health Officers, Environmental Health Technicians and Field Orderlies) who made up teams for supervisors of spraying teams were followed into the field as malaria spraying activities were being conducted, recruited into the study and were interviewed. District Health Executive members (District Medical Officer, District Environmental Health Officer, District Accountant, District Nursing Officer, District Pharmacy Technician and District Health Services Administrator) and the District Health Promotion Officer found on duty were interviewed as primary respondents as well. The Provincial Epidemic Diseases Control Officer, Provincial IRS Co-ordinator, District Environmental Health Officer and the Provincial Malaria Field Officer were conveniently sampled as key informants.

Using Epi Info™ version 3.5.1, StatCal function, assuming 42,8% of the participants will report late disbursement of funds as reason for poor program performance [11], assuming that the combined provincial total population of environmental health workers and members of the district health executive are 53, with the worst acceptable rate of 10% and at 95% confidence interval, a minimum calculated sample size of 32 primary respondents was to be interviewed.

**Data collection:** an interviewer administered questionnaire was used on primary respondents and district managers to get information on demographics of the health workers. A set of questions targeted on knowledge and awareness of respondents on malaria and pre-elimination processes. The second section dealt with sources of funding availability and adequacy of program inputs, role of partners in IRS as well as any activities that are done to supplement IRS budget. The last section of the questionnaire probes for the reasons for not achieving program targets.

Key informant interviews were guided by interviewer administered questionnaires which captured demography, knowledge on malaria pre-elimination, program inputs, funding and reasons for poor performance of the program.

A checklist was used to collect information on availability of resources, resources management strategies in place, equipment in place, shortages that were experienced during the program and storage and issuing of goods procedures. Part of the checklist looked at the plans for IRS, Epidemic Preparedness and Response (EPR), malaria surveillance and community involvement at district level. Completeness and timeliness of data was measured while stability of the system was evaluated. A desk review of available IRS records from 2011 to 2012 season was done to verify plans, quantification of inputs including data quality.

**Data management and analysis:** data was entered into Epi-InfoTM version 7, cleaned and checked for errors. This software was used for analysis to generate frequencies, means graphs and to calculate chi-square tests for trends.

**Ethical considerations:** all participants were treated with respect, written and informed consent was sought from the study participants. Confidentiality was maintained throughout the study, names of participants were not used and questionnaires were kept in a lockable secure cabinet during and after the study. No rewards or coercion of study participants was done throughout the study. Permission to conduct the study was sought and granted from the Provincial Medical Director Matabeleland South, District Medical Officers in the 5 districts and the Health Studies Office.

## Results

We interviewed 37 primary respondents, 68% were males and 32% females. Sixty-eight percent were direct program implementers from the environmental health department. The median age in service for the primary respondents was 5.5 (Q1=1, Q_3_=10.5) years. We also recruited 5 key informants into the study, all were males and their median age in service was 14 (Q_1_= 11; Q_3_= 24) years.

All 5 districts had plans for IHRS but without specific plans for the malaria pre-elimination. Surveillance and malaria trends analysis using available data was being done, however all the districts had no copies of the emergency preparedness plans. There were no Information Education and Counselling (IEC) materials with pre-elimination of malaria. Community and stakeholder sensitization meetings were cascaded from provincial, district and then to ward level.

The province was allocated 65% of the fuel they requested and thus there was not enough fuel for total coverage and incidentals such as call backs. There were 10 functional motorcycles from a request of 20. The province had 100 spray pumps and 62% were functional while spray-pump spares were not available. There were no chairs and torches for use in the camps. There were no respirators and belts for the spraymen however the rest of the protective clothing was supplied though not in adequate quantities. There were 32 four-man tents, 30% of them were worn out. The districts received funds that were less than their budgeted requests. Beitbridge received 87% ($26 520, 00) of requested budget while the rest of the districts received equal disbursements of $14 130, 00 equivalent to 86% of requested budget. 97.3% (36/37) of primary respondents said funding falls far below requested funds and 24% (9/37) of the primary respondents said their district do supplement funds for IHRS through building inspections. Procurement of insecticides is done centrally by the National Malaria Control Program. All the key informants (n=5) and the majority of the primary respondents (81%) knew insecticides are procured at national level.

The Global Fund was reported as the main funder of the program by all key informants and 81% of primary respondents. All key informants and 2.7% of primary respondents cited the Health Services Fund (HSF). The Health Services Fund (HSF) is used to buy food provisions during the commencement of spraying before the allowances for the spray teams are received from NMCP.

One partner (World Vision) was reported by 14% of primary respondents and 2 key informants to be assisting in some districts. World Vision assists the program mainly with trainings (35%), procurements of provisions (22%), provision of IEC materials (19%) and with transport (16%) while also helping with community mobilization (3%) as reported by primary respondents.

The majority of primary respondents (57%) said there is inadequacy of spraymen to conduct IHRS in the province. However all the key informants said the province had enough spray-men. According to the guidelines from records review, spraymen are adequate while all districts had 100% trained level 3 supervisors and at least 78% trained level 2 supervisors.

Spraying started late in the first week of October 2011 and was completed late on the 28^th^ of December 2011. On paper spraying was delayed, and thus coverage was below expected targets (Table 1). Data is captured, analyzed and reported to the province by data managers that are stationed in IRS camps, it was 100% complete. A total of 8 different forms that include daily spraymen log books are were inspected and they were completely filled. The province expects 3 reports from the districts and these were sent on time and this includes the final IHRS reports.

**Table 1:** IHRS coverage in districts conducting IHRS in Matebeleland South Province 2011

District	Targeted rooms	Rooms sprayed	% rooms sprayed	Targeted population	Population protected	%population protected
Beitbridge	54352	46024	85	93938	82607	88
Bulilima	30698	27367	89	40290	38987	97
Gwanda	24167	21275	88	33529	30230	90
Mangwe	11808	10857	92	15068	12867	85
Matobo	17560	15812	90	20553	18677	91

Mangwe district managed to spray all the 7 wards, while Gwanda, Bulilima and Matobo managed to spray all the designated 8 wards. In Beitbridge, they sprayed 11 out of the 15 targeted wards. However, not all households were sprayed even in those districts where all wards were covered (Table 1). The provincial IHRS coverage for 2011 was 84%, which is below the set target of 95%.

The main challenges reported by the primary respondents why the IHRS is not attaining its targets were; delays in starting spraying (67%), food shortages for spraymen (60%) and failing to complete spraying (41%). All key informants blamed late delivery of inputs and poor state of IHRS equipment for not meeting targets. In the field that 3 teams relied on one motor-cycle for warning. This affects information dissemination as door-to-door warning was not feasible. Motorcycles were made available by the districts but they were not serviced and could not be used by the IHRS teams.

All the key informants and 92% of primary respondents stated that there were problems in sourcing spares for the spray pumps and thus they could not service their pumps in the camps. There has been a shift from using the Hudson pump to the Micronair. The Hudson pump had spares that were easily accessible but the new Micronair pumps have no spares available. DDT is reported to have bigger granules and they block the pores of the strainers for the Micronair pumps, thus more blockages and broken down pumps are being experienced by the sprayers.

Community mobilization was reportedly being done mainly by the environmental health department (62%), while 27% of primary respondents said it was inter-departmental. Only 8% said the Health Promotion department was responsible for community mobilization. In the field, program implementers reported that the acceptance of DDT was poor. Some villagers refused for DDT to be sprayed on their walls as it leave them dirty with “whitish” stains. Some were afraid of the harmful effects of DDT.

The province has no specific camp sites built for IHRS and are using makeshift camps at the chosen rural health centres. There should be evaporation ponds that are fenced off and with floors made of impervious surfaces. Currently they are using makeshift evaporation ponds that liquid DDT waste seeps on to the ground while some of it is kept in plastic containers and the plans for its disposal are not clear. Solid DDT waste is kept under lock and key at the camp sites while awaiting collection for incineration.

All key informants were knowledgeable on malaria pre-elimination phases, plans and program re-orientation. They were all trained in program re-orientation, program targets and implementation including monitoring and evaluation of the malaria pre-elimination. Primary respondents were divided into direct program implementers (Environmental Health Officers, technicians, field orderlies) and non-direct program implementers, the District Health Executive members) and their knowledge was assessed (Table 2). Generally direct program implementers knew more that indirect program implementers but the knowledge trend was not statistically significant (p-value=0.4) (Table 2). Primary respondents knowledge among direct and indirect program implementers was aggregated, tallied and measured against a Likert scale from very poor (1) to excellent knowledge (4). 28/29 direct program implementers and 5/8 indirect program implementers had knowledge >2 Likert score respectively.

**Table 2:** Knowledge assessment on pre-elimination for primary respondents, Matebeleland South Province, 2012

Knowledge on	Direct program implementer	
	Yes n=29	No n=8
Province with pre-elimination plans	28	5
Coverage targets	20	3
Slide positivity	28	8
Malaria notification	26	6
	p=0.406	Chi=0.69

Sensitization workshops, intensified health education in the clinics and sending posters into the community were mentioned by both key informants and primary respondents as possible ways of educating the community and health workers on malaria pre-elimination. This should include the use of DDT and its advantages. All 5 key informants alluded to improved community participation, 4/5 was advocating for more resources from treasury and 3/5 needs to see program re-orientation as possible strategies that will enable the possible implementation of the malaria pre-elimination plan.

## Discussion

Funding for the IHRS program in Matebeleland South was inadequate, thus the challenges and possible solution revolves around funding gaps. Successes in malaria elimination are credited to intense national commitment to achieving zero incidence of infection together with the support from partners [6]. Lack of treasury capacity to fund the program leading to much reliance on the Global Fund to support the program is contributing to the delays. Furthermore, the allocated budget falls far short of requested budgets by the province to effectively run the program. In a study done in Mozambique, it was noted that there was an increase in the supply of malaria control commodities while there still were challenges in the procurement of IRS commodities [12]. In 2006 there were delays in procuring spray pumps, chemicals and other logistics led to untimely implementation of the IRS program in Zimbabwe, Zambia and Mozambique [12, 13].

Shortages of fuel led to a situation that there were no call-backs and if a household or village is missed due to competing programs such as funerals, village meetings or other donor food program then the missed households will not benefit from IRS. Motorcycles were availed to the program but they were not roadworthy and the program ended up having 3 teams sharing one motorcycle for warning. Thus there is need for the NMCP to set aside a budget for servicing and repair of motorcycles otherwise resources and time are wasted as the communities would not be aware of spraying activities taking place.

In a study done in Swaziland by Hlongwana et.al, they found out that there is need for improving the availability of information through the preferred community channels, as well as professional health routes [14]. Community mobilization is not well co-ordinated in the spraying districts. The Provincial Health Promotion Office should be on the forefront to coordinate awareness campaigns but they cite financial challenges as they are not given a budget by the NMCP. This then leaves the Environmental Health department to do health education and promotion. Apart from stakeholder sensitizations that are taking place, there should be efforts thus to improve the way program messages are conveyed to the community so as to improve on overall acceptance of the program.

Public officials need to mobilize funding and advocacy to assure political commitment and continuous funding [9]. There is also need for non-governmental organizations working with the Global Fund (such as Population Services International) to improve their visibility in Matebeleland South Province in collaboration with the Ministry of Health so that malaria pre-elimination messages are conveyed to the community through road shows and dissemination of IEC materials.

All the participants that were part of the study had not been trained on monitoring and evaluation of the malaria spraying program; therefore there is need for training and re-orientation on the goals and implementation of malaria pre-elimination in Matebeleland South Province. Program re-orientation is the key strategy where health service staff is reoriented to the new goals of malaria elimination [9]. It involves strengthening the health information system and improving the effective coverage of health interventions such as vector control and prevention including monitoring and evaluation [9, 10]. These messages should address the reintroduction of DDT explained to the community and the health workers especially on the safe use and benefits of DDT as there are fears, myths and misconceptions about the insecticide.

The province with the support of the NMCP has to commit resources in construction of malaria spraying camps that will have proper infrastructure which will contain fenced off evaporation ponds where DDT (both solid and liquid waste) will be opened, diluted and washed without seeping to contaminate the environment. Safety of use among spray operators, transportation, storage use and disposal of DDT has to be put in place by a country. Monitoring and evaluation is necessary for measuring the overall success of the IRS program using DDT [10].

## Conclusion

We can conclude that Matebeleland South Province has failed to achieve Malaria pre-elimination IRS coverage set targets in the years 2011 and 2012. There are financial and logistical challenges leading to delays in supply of program inputs, recruitment and training of spraymen. The province has no proper structures for camping and safe disposal of DDT waste and is currently using makeshift structures. Funding should be mobilised to cover aspects of health education, procurement of inputs and trainings. Thus it was recommended that there be training for health workers on malaria program re-orientation, to engage partners working with the Global Fund in the component of health promotion and come up with a provincial malaria pre-elimination health education and promotion plan. A budget for servicing of vehicles is strongly advocated for while construction of appropriate IRS camping facilities should be done. Further studies should be done to evaluate the effects of DDT in the communities and the health workers who handle.
